# Detection of Caries Around Resin-Modified Glass Ionomer and Compomer Restorations Using Four Different Modalities In Vitro

**DOI:** 10.3390/dj6030047

**Published:** 2018-09-16

**Authors:** Tamara Abrams, Stephen Abrams, Koneswaran Sivagurunathan, Veronika Moravan, Warren Hellen, Gary Elman, Bennett Amaechi, Andreas Mandelis

**Affiliations:** 1 Quantum Dental Technologies Inc., Toronto, ON M6B 1L3, Canada; tabrams@uoguelph.ca (T.A.); konesh@thecanarysystem.com (K.S.); 2 VM Stats, Toronto, ON M5A 4R3, Canada; vmoravan@vmstats.ca; 3 Cliffcrest Dental Office, Toronto, ON M1M 1P1, Canada; wmph@rogers.com (W.H.); garyelman@sympatico.ca (G.E.); 4 Department of Comprehensive Dentistry, University of Texas Health Science Center, San Antonio, TX 78229-3900, USA; amaechi@uthscsa.edu; 5 Center for Advanced Diffusion Wave and Photoacoustic Technologies (CADIPT), Department of Mechanical and Industrial Engineering, University of Toronto, Toronto, ON M5S 3G8, Canada; mandelis@mie.utoronto.ca

**Keywords:** caries, resin-modified glass ionomer, compomer, PTR-LUM, DIAGNODent, caries around restoration margins, SPECTRA, ICDAS II, caries detection

## Abstract

The aim of this study was to evaluate the ability of visual examination (International Caries Detection and Assessment System—ICDAS II), light-emitting diodes (LED) fluorescence (SPECTRA), laser fluorescence (DIAGNODent, DD), photothermal radiometry and modulated luminescence (PTR-LUM, The Canary System, CS) to detect natural decay beneath resin-modified glass ionomer (RMGIC) and compomer restorations in vitro. Twenty-seven extracted human molars and premolars, consisting of 2 control teeth, 10 visually healthy/sound and 15 teeth with natural cavitated lesions, were selected. For the carious teeth, caries was removed leaving some carious tissue on one wall of the preparation. For the sound teeth, 3 mm deep cavity preparations were made. All cavities were restored with RMGIC or compomer restorative materials. Sixty-eight sites (4 sites on sound unrestored teeth, 21 sound sites and 43 carious sites with restorations) were selected. CS and DD triplicate measurements were done at 2, 1.5, 0.5, and 0 mm away from the margin of the restoration (MOR). SPECTRA images were taken, and two dentists provided ICDAS II scoring for the restored surfaces. The SPECTRA data and images were inconclusive due to signal interference from the restorations. Visual examinations of the restored tooth surfaces were able to identify 5 of the 15 teeth with caries. In these situations, the teeth were ranked as having ICDAS II 1 or 2 rankings, but they could not identify the location of the caries or depth of the lesion. CS and DD were able to differentiate between sound and carious tissue at the MOR, but larger variation in measurement, and poorer accuracy, was observed for DD. It was concluded that the CS has the potential to detect secondary caries around RMGIC and compomer restorations more accurately than the other modalities used in this study.

## 1. Introduction

One of the major reasons for the replacement of restorations is secondary caries or caries around the restoration margins [1,2]. Caries detection around restoration margins including RMGIC and compomers is a major challenge in clinical practice. Compomers and RMGIC restorations release fluoride which may have promising results in caries prevention around restoration margins [3,4,5,6]. Systematic reviews show significant decreases of new lesions around RMGIC restorations compared to amalgam [7] and composite restorations [8,9]. However, the challenge is to detect these lesions early; before caries has destroyed more tooth structure and larger, more invasive replacement restorations are required [1].

The detection of secondary caries in the early stages of the disease process is challenging [10], especially, with current detection methods which included visual examination, use of explorers or blunt probes, radiography, and or fluorescence-based devices [11]. Visual or visual-tactile examination, using explorers or probes, often in combination with bitewing radiographs, are the most commonly used technique in clinical practice for caries detection [12].

The International Caries Detection and Assessment System (ICDAS II) was introduced in 2009 to assist in visual ranking of caries [13,14,15,16]. The surface appearance of restorations with secondary caries is considered similar to primary caries lesions so the ICDAS II criteria can be used for ranking secondary caries around restorations [17,18]. Research has shown that the ICDAS II presents good reproducibility and accuracy for in vitro and in vivo detection of primary caries lesions at different points in the disease process [18,19,20].

Laser fluorescence (DIAGNODent 2095 (LF), KaVo, Biberach, Germany) has been used as an aid in detecting caries beneath restorations [12,21]. In 2006, a new laser fluorescence device (DIAGNODent 2190 (LFpen), KaVo) was introduced to aid in the detection of occlusal and interproximal caries. The LFpen, using a low powered 655 nm wavelength diode laser, can analyze and quantify the fluorescence emitted from bacterial porphyrins and other chromophores [22,23]. In vitro studies have demonstrated that LF can detect caries at the margins of amalgam restorations, but amalgam overhangs and stain reduce the sensitivity of this method [24,25,26].

The SPECTRA Caries Detection System (SPECTRA Air Techniques Melville, New York, NY, USA) also uses fluorescence technology as well. Light-emitting diodes (LED) projects 405 nm wavelength of light onto the tooth surface causing cariogenic bacteria to fluoresce red and healthy enamel to appear green [27,28]. SPECTRA software then quantifies the fluorescence on scale ranging from 0 to 5 [29]. SPECTRA also captures the fluorescence from bacterial porphyrins [28,30,31]. Studies have shown the ability of SPECTRA to detect caries on unrestored occlusal surfaces [32,33,34,35] but the detection around restoration margins or beneath sealants has been more challenging [36,37,38].

The Canary System (Quantum Dental Technologies, Toronto, ON, Canada) using a 660 nm <50 mW, pulsed laser, combines laser photothermal radiometry (PTR) and modulated luminescence (LUM) amplitude and phase signals to detect and assess caries [39]. Pulses of laser light focused on a tooth cause, the tooth to “glow” or luminesce (LUM) and releases heat (PTR). The system analyzes the response of the re-emitted radiation (luminescence or LUM) and the thermal behavior of the emitted infrared photons (PTR) to provide information about the status of the tooth’s crystal structure [39]. The CS measures both the amplitude and phase delay of the PTR and LUM signals and then converts these signals into a measurement or Canary Number (CN). These pulses of laser light can detect caries lesions up to 5 mm below the tooth surface [39,40,41]. As a caries lesion increases in volume there is a corresponding change in the PTR and LUM signals [40]. The heat is confined to the region with crystalline disintegration (dental caries) increasing the PTR and decreasing the LUM signals [42]. During remineralization, the enamel prisms start to reform their structure and the thermal and luminescence properties begin to revert towards those of healthy tooth structure [43,44,45,46].

This study assessed the ability of four caries detection systems to detect secondary caries beneath the margins of RMGIC and compomer restorations. This in vitro model does simulate a clinical situation where restoration margins are intact but secondary caries is present beneath one section of the restoration.

## 2. Materials and Methods

### 2.1. Study Design

Following the approval of the Institutional Review Board (IRB Approval: HSC20080233N) of the University of Texas Health Science Center at San Antonio (UTHSCSA), freshly extracted unidentified human teeth appropriately disposed in various clinics of the UTHSCSA Faculty of Dentistry, were collected and examined. Twenty-seven extracted human molars and premolars, consisting of 12 visually sound/healthy teeth and 15 teeth with natural cavitated lesions were selected. Teeth with open caries lesions where selected, where the caries could be restored by the placement of an RMGIC or compomer restoration. Surface debris and stain was removed from the teeth, but the caries lesions were not touched. The teeth were stored in distilled water to avoid dehydration, using the protocol established in our earlier studies [39,47,48]. Before examination each tooth was removed from the vial, rinsed thoroughly with distilled water for 20 s and air-dried for five seconds.

Two healthy teeth were set aside as sound healthy samples. They were used to confirm that storage media and sample handling did not alter readings with the various modalities. These teeth were scanned at two spots on each tooth. Of the remaining 25 teeth, 10 teeth were identified as healthy/sound and 15 teeth had visible caries lesions.

A dentist selected the smooth surface to be restored on the tooth samples. A standard RMGIC/compomer preparation was done using high speed handpiece bur to remove enamel. A slow speed hand piece with round carbide bur was used to remove dentin and caries. The cavity preparation on the sound samples was at least 3 mm in depth. On the samples with caries, the carious tissue was removed from the tooth, except on one wall. On that wall, the caries and demineralized enamel was removed from the preparation margin, but caries was left at least 1 mm below the tooth surface with the caries covering at least 3 mm width of the preparation wall. All measurements, during the cavity preparation, were done with a periodontal probe (Williams Periodontal Probe PW6 Hu-Friedy, Chicago, IL, USA).

Three restorative materials were used:

- Dyract eXtra Dentsply Refill Compules Shade A2 Lot., 1608001074; Expiry August 2018 (3M ESPE St. Paul MN., USA).
- Ketac Nano 3M Shade A2 Ref. 3304A2 Lot., N733107; Expiry May 2017 (Dentsply DeTrey GmbH, Konstanz, Germany).
- Compoglass F Ivoclar Vivadent Refill: Shade 140/A2 Lot., V19970; Expiry October 2018 (Ivoclar Vivadent AG, Schaan, Liechtenstein).

When the preparations were completed, the teeth were photographed on all surfaces. Standard bonded compomer/RMGIC technique was used for the placement of the restorations. The cavity preparation was etched using 37% phosphoric acid gel (Temrex Gel Etch, Temrex Corporation. Freeport, NY, USA) for 30 s. The teeth were rinsed with water for 30 s to remove the phosphoric acid gel and then air-dried for 30 s. Bond1 Primer/Adhesive (Pentron Clinical Technologies, Orange County, CA, USA) was applied inside the cavity preparation to bond restoration. The bond was cured with a dental curing light (Demi-Ultra LED Curing Light Kerr, Orange County, CA, USA) for 20 s. The restorations were then placed in 3 mm depth increments and light cured. Any excess material on the surface was removed. After the restorations were placed the teeth were put back into distilled water for storage for 1 month.

Photographs were taken of all the tooth surfaces after placement of the restorations. On each photograph a section of the restoration was selected for examination. On samples with caries beneath restoration, a section of the carious margin was selected for examination and marked on the photographs.

On the ten healthy/sound teeth, a total of 21 areas were examined and on the fifteen teeth with caries a total of 43 areas were examined. On the 10 sound restored teeth, 21 sites (8 sites for Dyract eXtra, 7 sites for Ketac Nano and 6 sites for Compoglass F) were examined. On the 15 carious sample teeth, 43 sites (15 sites for Dyract eXtra, 15 sites for Ketac Nano and 13 sites for Compoglass F) were examined. In total there were 23 spots scanned with CN and DD on Dyract eXtra restorations, 22 spots scanned on Ketac Nano restorations, 19 spots scanned on Compoglass F restorations and 4 sites on standard teeth. In summary, 68 sites (4 sites on sound unrestored teeth, 21 sound sites with restorations; 43 carious sites) were examined with CN and DD.

**Figure 1:**
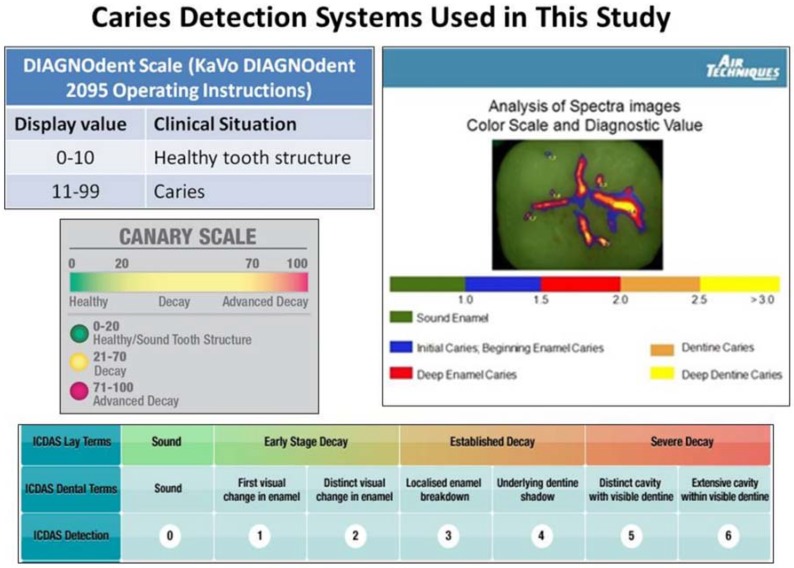
Caries system detection scales for devices used in this study.

A technician, not involved in restoration of the teeth, took DD and CS measurements at the MOR, 0.5 mm, 1.5 mm and 2.0 mm away from the MOR of the RMGIC and compomer margins. Three readings were taken at each position and the measurements were recorded. The means and standard deviation for each measurement taken at each position were calculated. The measurement scales for the various caries detection systems, used in the study, are shown in Figure 1.

### 2.2. ICDAS II Visual Examination

Two dentists, trained in using the ICDAS II visual scoring system [49], scored each tooth surface with a restoration independently. The ICDAS II criteria used in the study were:

0 Sound tooth surface;
1 First visual change in enamel (on a dry tooth surface);
2 Distinct visual change on enamel surface (on a moist and dry tooth surface);
3 Localized enamel breakdown due to caries with no exposed dentin or shadowing beneath the tooth surface;
4 Dark shadow beneath the tooth surface from dentin;
5 Distinct cavity with visible dentin;
6 Extensive distinct cavity with visible dentin and more than half of the surface involved.

All ICDAS examinations were conducted in a dental operatory using a dental operatory light. No visual aids such as microscopes or magnifying loupes were used. Where there was disagreement between the clinicians’ scores, the tooth surfaces were re-examined by both clinicians at the same time and agreement was reached on the ICDAS score (consensus score). The clinicians’ scores and consensus scores were recorded, and the consensus scores were used in this study.

### 2.3. SPECTRA Caries System Examination

SPECTRA recorded an image of each tooth surface being examined using SPECTRA Imaging software and stored it on a computer. A 10-mm distance spacer and the SPECTRA handpiece disposable camera covers were used (AIR TECHNIQUES, Melville, New York, NY, USA) during image acquisition.

### 2.4. DIAGNODent Examination

DIAGNOdent Classic (KaVo Dental model 2095, Biberach, Germany) was used following the manufacturer’s operating instructions. Probe “A” was used for measurements at various distances from the restoration margin. Before examining each tooth, DD was calibrated with the calibration disc. The tooth was air-dried for five seconds and the tip of the DD was placed perpendicular to the examination site. Three measurements were recorded for each site and the mean peak value was calculated.

### 2.5. The Canary System Examination

The CS was used following the manufacturer’s operating instructions. The CS was calibrated before each tooth was scanned. The tooth was air-dried for five seconds and the cone of the disposable plastic tip was positioned perpendicular over the examination site and a measurement was taken. Three measurements were taken at each site and recorded. The mean value was calculated.

### 2.6. Blinding of the Participants in This Study

Several actions were taken to blind the participants in this study. One dentist selected the tooth samples for inclusion in the study and placed the various restorations. A technician examined the tooth surfaces using CS, DD and SPECTRA. Two clinicians did the visual assessment of the surfaces using ICDAS II criteria. The dentist who placed the restoration was the only study participant that knew which teeth had caries beneath the restorations. Statistical analysis was done by a statistician not involved in the sample selection or examination.

### 2.7. Statistical Analysis

Since the teeth had been pre-selected as sound and carious before examination with the various systems, they were divided into these two groups for analysis. Sensitivity and specificity analysis were performed on the data collected using CS, SPECTRA, ICDAS II and DD.

Three measurements using CS and DD were conducted on each tooth spot, as per the protocol. Intra-operator repeatability analysis was done for the 3 CS and DD readings on each spot. The intraclass correlation (ICC) was used to measure intra-rater reliability of individual scans by spot scanned. The ICC was calculated using two-way random effects model, under the absolute agreement definition.

Descriptive statistics, including means, standard errors, standard deviations, and 95% confidence intervals were calculated for all measurements. The means for CS and DD were analyzed at the MOR, 0.5 mm, 1.5 mm, and 2 mm away from the restoration margin. Differences between means of restored sound and restored carious teeth were tested with two-sample *t*-tests, after use of Levene’s test for equality of variances to determine if separate or pooled variances were called appropriate. Any testing between means involving unrestored teeth was done using Wilcoxon signed rank test because of the small sample size. All *p*-values were two-sided and statistical significance was determined using the traditional *p*-value of <0.05. The sensitivity and specificity (with 95% CI) were done for all measurements and analyzed overall and by restorative materials. The intra-operator repeatability was assessed by calculating the intraclass correlation coefficient (ICC). 

All analysis was done using R software (version 3.4.3, R Core Team, Vienna, Austria) [50]. The T-test and Wilcoxon signed rank test was calculated using R software functions “t.test” and “wilcoxon.test” respectively, in R package “stats”. Levene’s test for equality of variances was calculated using function “LeveneTest” in R package “car”. Confidence intervals for sensitivity and specificity were calculated using Wilson method, using function “binom.confint” in R package “binom” [50]. The intraclass correlation coefficient (ICC) was calculated using function “ICC” in R package “IRR” [50].

## 3. Results

Two clinicians using ICDAS II ranking for visual inspection of the RMGIC/compomer margins were only able to locate 5 teeth with caries beneath the restoration margins. On these 5 teeth the agreed ICDAS ranking were; 3 teeth at ICDAS 1 (2 teeth restored with Compoglass F and 1 restored with Dyract eXtra) and 2 teeth at ICDAS 2 (2 teeth restored with Ketac Nano). For healthy samples, the examiners ranked five surfaces as ICDAS 1 and the rest were ranked as ICDAS 0 or healthy. The ICDAS 1, rankings, on healthy teeth, were associated with 2 Dyract eXtra restorations, 1 Ketac Nano restoration and 1 Compoglass F restorations and one on the control tooth. All the other RMGIC or compomer margins on both carious and sound samples were ranked as ICDAS 0 (healthy). The ICDAS II, examination sensitivity and specificity were 0.35 and 0.52, respectively. Visual ranking using ICDAS II did not appear to be an accurate method for detecting caries beneath restoration margins, in this study. There appeared to be no correlation with lesion detection and restorative material in this study when using ICDAS II ranking.

**Figure 2:**
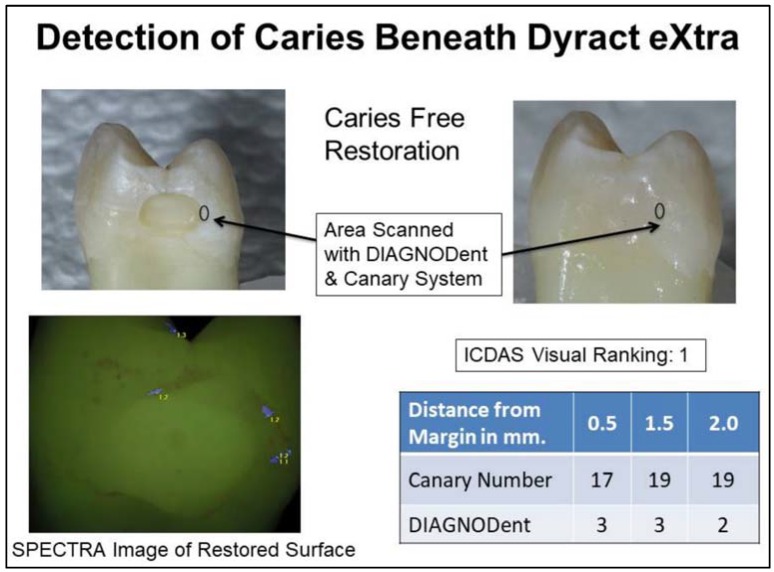
Examination of caries free margin of a Dyract eXtra restoration.

**Figure 3:**
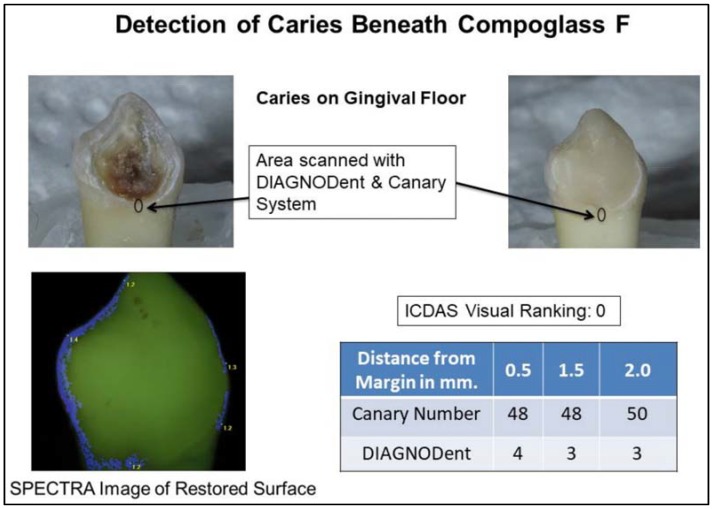
Detection of caries beneath Compoglass F restoration margin.

The SPECTRA images of the RMGIC or compomer restoration all appeared as green. At times the color was slightly darker than the surrounding tooth structure. Near the margins of some of the restorations, there were very thin blue or red lines (see Figure 2 and Figure 3). At times these lines were associated with the edges of the tooth surface or with stain on the surface. The majority of tooth surface examined, appeared green, indicating sound enamel, even if caries was present beneath the MOR. The compomer and RMGIC had very low reflectivity so SPECTRA was not able to accurately measure fluorescence around the MOR. This study found that the SPECTRA data and images were inconclusive due to signal interference from the restorations.

Table 1 shows the mean CN and DD readings on the compomer or RMGIC margin and at various distances from the margins of the restoration. At the MOR, the CN from teeth with caries beneath MOR were 45 ± 15.7. On healthy MOR the CN was 20.1 ± 5.7. The CN at 0.5, 1.5 and 2.0 mm away from the restoration margin on sound samples remained below 20 indicating no caries present. However, on teeth with caries beneath the restoration margin, the CN measurements at 0.5, 1.5 and 2.0 mm away from the margin gave means ranging between 45.7 and 52.2, indicating that there was caries beneath the restoration margin. The CN on caries samples did not drop significantly at 2 mm away from the restoration margin. Difference between means of sound and carious samples was statistically significant, at *p* < 0.001, at every distance from the restoration margin. Table 2 shows the sensitivity/specificity for sites at 2.0, 1.5, 0.5, 0 mm from the MOR which ranged from 0.91–1.0/0.71–0.93 for the CS.

DD gave readings of 17.2 ± 10.6 at the MOR in sound teeth. This dropped to 5 ± 3.4 at 2 mm from the restoration margin. Difference between means of sound and carious samples was not statistically significant at any distance from the margins of the restorations. On teeth with caries beneath restoration margins the DD reading was 19.5 ± 18.7 and dropped to 8.6 ± 8.81 at 2 mm away from the restoration margin. The sensitivity/specificity for sites at 2.0, 1.5, 0.5, on the margin ranged 0.19–0.7/0.14–0.93 for DD (Table 2).

From examining the data and looking at practical applications in clinical practice [38], it appeared that examining the restorations at 0.5 mm from the restoration margin provided the most accurate data for clinical assessment. Standard teeth scanned at 0.5 mm from the margin, yielded CN mean (SD) of 16.8 (2.2). This was similar to CN at 0.5 mm from margin for healthy/sound teeth 18.1 (3.3), *p*-value = 0.25, but differed from carious teeth 46.8 (18.7), *p*-value = 0.003. Using DD, standard teeth yielded 4.5 (0.6), compared to sound teeth 10 (7.3) and carious teeth 11.7 (15.4), *p*-values were 0.095 and 0.018. Wilcoxon signed rank test were used in all comparisons of standard teeth to sounds or carious teeth.

When measuring 0.5 mm away from a restoration placed on a healthy/sound tooth the DD reading was approximately 10, at the top end of the range for healthy teeth. When measuring around restorations with caries the DD measurements (Table 1) rose to around 12 indicating caries was present but not providing an indication of the size or extent of the lesion. The overall sensitivity and specificity for detection of caries around RMGIC and compomer restorations was best when using CS at 0.5 mm from the restoration margin (Table 2).

An analysis was done on the potential impact that the RMGIC or compomer material had on the ability to detect caries beneath the respective restoration margins. Table 1 shows the data for CS and DD. When scanning with CS around the 3 materials margins with caries beneath the margins, (Table 1) all had CN in the range between 30 and 68 indicating that caries was present. On restorations placed in healthy/sound teeth the CN remained below 20 indicating no caries was present. Each restoration material type showed statistically significant differences in CN means, at *p* < 0.001, between healthy restored and carious teeth. Since the scanning was done 0.5 mm from the restoration margin it is possible that the restorative material might have contributed to the size of the CN. This study did not produce standard sized lesions, so one could not assess the impact of the restorative material on CN. Even if the material did increase the CN it did not drive the number over the healthy range when scanning the margins of restorations placed in healthy/sound teeth.

In examining the DD reading around the three different materials (Table 1), the Dyract-Xtra and Ketac Nano restorations did show an increase between restorations placed in healthy and carious teeth, but the differences were not statistically significant, *p* = 0.073 and *p* = 0.496 respectively. With the Dyract eXtra restorations, beyond healthy margins measured just under 5 and the margins with caries beneath them, were just below 10. Although the readings were different on the DD scale, these measurements indicated that there were no caries on restorations placed over caries lesions. Ketac Nano restorations placed on healthy/sound teeth had DIAGNODent readings just above 10 indicating caries and the restorations placed over caries had readings around 15. Compoglass F restorations placed on healthy teeth had DD readings of 14.8 ± 6.5 and restorations placed over caries had readings of 6.6 ± 2.25. In this situation, DD was not able to accurately identify caries lesions.

The overall intra-operator repeatability [50], when using CS or DD, was high (Table 3), for both systems.

## 4. Discussion

The longevity of restorations depends upon many factors including materials used, type of restorative procedure, size and depth of the lesion, patient parameters such as caries risk, oral hygiene, operator variables and other local factors. Some of the major reasons for restoration failures are secondary caries, restoration or tooth fracture, marginal deficiencies, wear, and postoperative sensitivity [2]. The development of caries adjacent to existing restorations is a multifactorial problem that is difficult to study in vivo, due to human variability and the time required for identifiable lesion to form [51]. This in vitro model does not exactly emulate what would occur, in vivo. In clinical practice a restoration is placed into a cavity preparation that has sound, caries-free walls. This in vitro model was chosen to simulate caries on the wall of a restoration which would develop months or years after the initial placement of the restoration. The study was designed to see if various caries detection systems could detect caries beneath the visibly intact margins of RMGIC and compomer restorations.

In clinical practice, visual or visual-tactile examinations (use of an explorer or blunt probe), often combined with bitewing radiography, are still the most common techniques for examining the marginal integrity of restorations [52]. Since the study involved examining visible smooth surfaces radiographs were not included. Visual changes adjacent to restoration margins such as discoloration, staining, or dentinal shading, may be caused by a lot of clinical factors; only one of them being secondary caries [53,54]. The two dentists using ICDAS II scoring for visual assessment, could only detects caries beneath the restoration margin in a few of the samples.

Fluorescence-based caries detection devices may encounter challenges in detecting caries around RMGIC and compomer margins. Some studies have found that measuring fluorescence may not be suitable for detecting caries around restoration margins due to false positive readings [25,55,56,57]. The CR Clinicians Report (March 2012), found that existing restorations may cause interference in readings from these devices [58]. Fluorescence-based technologies may not give any information about lesion size, volume or depth [59,60]. Scattering of the light and fluorescence caused by stain, plaque, organic deposits and surface features such as pits and fissures may prevent deep penetration of the light below the tooth surface. In this study, SPECTRA images were not able to detect caries beneath the restoration margins in vast majority of the tooth samples (Figure 2 and Figure 3).

DD is also a fluorescence-based device but uses a 660 nm wavelength which is not the wavelength used in SPECTRA. DIAGNOdent also does a point measurement so it was able to pick up some information from the tooth structure adjacent to the restoration margin with some interference from the restoration [61]. Overall DD was less consistently able to detect sound and carious margins. DD was not able to accurately identify sound or caries tissue beneath the margins of teeth restored with Compoglass F.

The CS can examine an area of approximately 1.5 mm in diameter and up to 5 mm below the tooth surface [42]. It provides a CN (ranging from 0–100) from an algorithm combining the PTR and LUM amplitude and phase measurements, which are directly linked to the status of the tooth’s structure being examined (Figure 1) [42]. A CN of less than 20 indicates healthy tooth structure [42]. A CN greater than 70 indicates the presence of a large lesion that may justify restoration [42]. CNs falling between 20 and 70 indicate the presence of caries or cracks that may require restoration or other preventive treatments-based upon further patient evaluation including caries risk factors [38,40]. If the caries is located beneath a healthy layer of enamel, the CS measures both healthy tissue and caries around and beneath the beam. The healthy tooth overlying the caries dampens the signal, decreasing the CN but keeping it above the CN healthy range [37]. In this in vitro study, The CS was able to examine the margins of the RMGIC or compomer restoration and up to 2 mm beyond the restoration margin and in the vast majority of the tooth samples, discern if there was healthy or carious tissue present beneath the MOR.

## 5. Conclusions

CS and DD were able to differentiate between sound and carious tissue at the MOR more accurately than ICDAS II and SPECTRA. DD had less reliability, larger variation in measurement and poorer accuracy for detecting caries when compared to CS. When scanning at 0.5 mm from the RMGIC or compomer restoration margin DD was not able to accurately detect caries or healthy margins. Therefore, CS has the potential to detect secondary caries around RMGIC and compomer restorations more accurately than visual examination with ICDAS II, SPECTRA or DD.

## Figures and Tables

**Table 1:** Canary number and DIAGNODent readings by distance from the margin of the restoration and by material.

Distance from the Margins of the Restoration	Canary Number			Peak DIAGNODent		
	(21–100 Denotes Caries)			(11–99 Denotes Caries)		
	Sound Teeth	Carious Teeth	*p*-Value ^1^	Sound Teeth	Carious Teeth	*p*-Value ^1^
	Mean (SD)	Mean (SD)		Mean (SD)	Mean (SD)	
**By Distance from Margin (All Materials)**						
At margin	20.1 (5.7)	47.7 (19.9)	<0.001	17.2 (10.6)	19.5 (18.7)	0.414
0.5 mm	18.8 (3.3)	46.8 (18.7)	<0.001	10 (7.3)	11.7 (15.4)	0.396
1.5 mm	19.3 (4.7)	45 (15.7)	<0.001	7.5 (5.9)	9.4 (7.8)	0.122
2 mm	18.3 (2.6)	52.2 (19.6)	<0.001	5 (3.4)	8.6 (8.8)	0.076
**Dyract eXtra**						
At margin	19.9 (6.3)	32.6 (12.5)	0.014	20.5 (11.7)	21 (18.5)	0.941
0.5 mm	17.1 (2.4)	31.8 (9.4)	<0.001	5.1 (1.6)	9.6 (6.6)	0.073
1.5 mm	17.4 (1.5)	37.2 (13.4)	0.003	4.9 (1.1)	7.7 (4.1)	0.076
2 mm	18.6 (1.3)	40.3 (12.2)	0.002	4.5 (1.6)	10.8 (13.6)	0.334
**Ketac Nano**						
At margin	19.2 (5.8)	63.2 (14.8)	<0.001	11.7 (6.4)	23.9 (24.9)	0.211
0.5 mm	18.6 (3.7)	63.7 (13.7)	<0.001	11.5 (9.1)	18.1 (24.4)	0.496
1.5 mm	18.7 (4.1)	56.7 (16)	<0.001	8.9 (7.7)	15.1 (11.1)	0.212
2 mm	17.8 (3.9)	68.4 (22.5)	<0.001	5.8 (5.2)	10.7 (6.5)	0.127
**Compoglass F**						
At margin	21.4 (5.7)	47.4 (19)	0.005	19.3 (12)	12.6 (4.6)	0.238
0.5 mm	21.3 (2.7)	44.5 (16.1)	<0.001	14.8 (6.5)	6.6 (2.5)	0.028
1.5 mm	22.7 (6.7)	42.2 (11)	0.001	9.4 (6.9)	5.8 (2.2)	0.097
2 mm	18.8 (0.5)	46.8 (9.4)	<0.001	4.4 (1)	4.5 (1.3)	0.93

^1^ Two-sample *t*-test.

**Table 2:** Sensitivity and specificity ICDAS II, SPECTRA, DIAGNODent and Canary System. For DIAGNODent and Canary System the sensitivity and specificity are given at various distances from the restoration margins.

Caries Detection System	Sensitivity (95% CI)	Specificity (95% CI)
ICDAS II	34.9 (22.4, 49.8)	52.4 (32.4, 71.7)
SPECTRA	34.9 (22.4, 49.8)	61.9 (40.9, 79.2)
Peak DIAGNODent at margin	69.8 (54.9, 81.4)	14.3 (5, 34.6)
Peak DIAGNODent at 0.5 mm from margin	30.2 (18.6, 45.1)	66.7 (45.4, 82.8)
Peak DIAGNODent at 1.5 mm from margin	19.5 (10.2, 34)	90.5 (71.1, 97.3)
Peak DIAGNODent at 2 mm from margin	18.8 (8.9, 35.3)	92.9 (68.5, 98.7)
Canary Number at Margin	97.7 (87.9, 99.61)	76.2 (54.9, 89.4)
Canary Number at 0.5 mm from margin	90.7 (78.4, 96.3)	81 (60, 92.3)
Canary Number at 1.5 mm from margin	95.1 (83.9, 98,7)	71.4 (50, 86.2)
Canary Number at 2 mm from margin	100 (89.3, 100)	92.9 (68.5, 98.7)

**Table 3:** Repeatability of DIAGNODent and Canary System measurements at 0.5 mm from the restoration margin.

Restorative Materials	Canary Number	Peak DIAGNODent
	ICC ^1^ (95% CI)	ICC ^1^ (95% CI)
All materials	0.99 (0.99, 1.0)	0.98 (0.98, 0.99)
Dyract eXtra	0.98 (0.97, 0.99)	0.89 (0.78, 0.95)
Ketac Nano	0.99 (0.99, 1.0)	0.99 (0.98, 1.0)
Compglass F	0.99 (0.97, 0.99)	0.96 (0.92, 0.98)

^1^ Intraclass Correlation Coefficient.
